# Gut microbiota and serum metabolomic alterations in modulating the impact of fecal microbiota transplantation on ciprofloxacin-induced seizure susceptibility

**DOI:** 10.3389/fmicb.2024.1403892

**Published:** 2024-06-19

**Authors:** Shangnan Zou, Yinchao Li, Qihang Zou, Man Yang, Huifeng Li, Ruili Niu, Huanling Lai, Jiaoyang Wang, Xiaofeng Yang, Liemin Zhou

**Affiliations:** ^1^Clinical Neuroscience Center, The Seventh Affiliated Hospital of Sun Yat-sen University, Shenzhen, Guangdong, China; ^2^Department of Neurology, The First Affiliated Hospital of Guangzhou Medical University, Guangzhou, Guangdong, China; ^3^Department of Basic Medicine, Guangzhou National Laboratory, Guangzhou, Guangdong, China; ^4^Department of Neurology, The First Affiliated Hospital of Sun Yat-sen University, Guangzhou, Guangdong, China

**Keywords:** gut microbiota, ciprofloxacin, seizure susceptibility, microbiota-gut-brain axis, fecal microbiota transplantation, untargeted metabolism, lithium pilocarpine

## Abstract

**Introduction:**

The gut microbiota and the microbiota-gut-brain axis have gained considerable attention in recent years, emerging as key players in the mechanisms that mediate the occurrence and progression of many central nervous system-related diseases, including epilepsy. In clinical practice, one of the side effects of quinolone antibiotics is a lower seizure threshold or aggravation. However, the underlying mechanism remains unclear.

**Methods:**

We aimed to unravel the intrinsic mechanisms through *16S* rRNA sequencing and serum untargeted metabolomic analysis to shed light on the effects of gut microbiota in ciprofloxacin-induced seizure susceptibility and lithium pilocarpine-induced epilepsy rat models.

**Results:**

We observed that ciprofloxacin treatment increased seizure susceptibility and caused gut dysbiosis. We also found similar changes in the gut microbiota of rats with lithium pilocarpine-induced epilepsy. Notably, the levels of *Akkermansia* and *Bacteroides* significantly increased in both the ciprofloxacin-induced seizure susceptibility and lithium pilocarpine-induced epilepsy rat models. However, *Marvinbryantia, Oscillibacter*, and *Ruminococcaceae_NK4A214_group* showed a coincidental reduction. Additionally, the serum untargeted metabolomic analysis revealed decreased levels of indole-3-propionic acid, a product of tryptophan-indole metabolism, after ciprofloxacin treatment, similar to those in the plasma of lithium pilocarpine-induced epilepsy in rats. Importantly, alterations in the gut microbiota, seizure susceptibility, and indole-3-propionic acid levels can be restored by fecal microbiota transplantation.

**Conclusion:**

In summary, our findings provide evidence that ciprofloxacin-induced seizure susceptibility is partially mediated by the gut microbiota and tryptophan-indole metabolism. These associations may play a role in epileptogenesis, and impacting the development progression and treatment outcomes of epilepsy.

## 1 Introduction

Epilepsy is a chronic neurological disease characterized by recurrent and transient brain dysfunction. It affects approximately 65 million patients worldwide and poses a substantial global health challenge (Fisher et al., 2014). Despite extensive research into the pathogenic mechanisms of epilepsy, its etiology remains unclear. Approximately one-third of patients with epilepsy who do not respond to currently available antiseizure medications develop drug-resistant epilepsy (DRE). Moreover, some daily factors, such as fatigue, hunger, alcohol or stimulant beverage consumption, insomnia, or infections can aggravate seizures. These observations underscore the complexity of epilepsy and the need for further exploration to understand its etiology and management.

Studies of the proposed concept of the microbiota-gut-brain axis (MGBA) have demonstrated its vital role in the pathological process of epilepsy (Yue et al., 2022). The gut microbiota (GM) is a complex group of symbiotic microorganisms (bacteria, viruses, and fungi) dwelling in the gastrointestinal tract, and is considered the second genome in humans (Ursell et al., 2012). MGBA facilitates communication between the brain and GM through various pathways. These include the autonomic and enteric nervous systems, immune system and neuroimmunity, neurotransmitters, short-chain fatty acids (SCFAs), spinal mechanisms, hypothalamic-pituitary-adrenal axis, and peptidoglycans, etc. (Cryan et al., 2019). Studies on the relationship between GM and epilepsy initially emerged with a ketogenic diet (Olson et al., 2018; Gong et al., 2021). Several clinical studies have shown substantial differences in the GM profiles among epilepsy patients, healthy individuals, and those with DRE (Arulsamy et al., 2020; Gong et al., 2020). The role of the GM in epileptogenesis and the development of epilepsy has been gradually confirmed in different epileptic animal models (Citraro et al., 2021; Mengoni et al., 2021; Mu et al., 2022b). The aforementioned evidence underscores the important role of the GM in epilepsy.

In clinical practice, it has been observed that the use of certain antibiotics may increase susceptibility to seizures, particularly within the β-lactam, fluoroquinolone families, fourth-generation cephalosporins, and carbapenems (Wanleenuwat et al., 2020). Ciprofloxacin (CPF) is an antibiotic belonging to the fluoroquinolone class that is effective against a wide range of bacteria. Previous studies have shown that quinolone antibiotics, including CPF, affect seizure susceptibility in various animal models and patients with epilepsy (Abdel-Zaher et al., 2012; Arafa et al., 2013; Cheraghmakani et al., 2021; Sivarajan and Ramachandran, 2023). When CPF is applied, fecal concentrations are high and CPF exerts a long-lasting effect on the GM (Zimmermann and Curtis, 2019). Previous studies on the mechanism of quinolone antibiotic-induced seizures have primarily focused on their potential to change neurotransmitter levels, disrupt neurotransmitter-receptor binding, have a chemical structure similar to that of epileptogens, and increase oxidative stress due to drug interactions (Ilgin et al., 2015; Wanleenuwat et al., 2020). However, the vital role of the GM has been neglected.

Therefore, the above-mentioned evidence predominantly examines the variations in GM between individuals with epileptic status and healthy controls, or among those undergoing different drugs or diet treatments. There is a notable lack of investigation into the therapeutic effects of GM modulation and the underlying mechanisms of the MGBA. Additionally, there has been a lack of recognition regarding the significant impact of CPF on the GM. Our study aims to combine the two aspects and fill this gap by focusing on elucidating the pathway of CPF induce seizure susceptibility through the GM.

To date, no studies have examined whether CPF increases seizure susceptibility from the perspective of GM and metabolite changes in animals. Recent studies have revealed that the GM is a major determinant of plasma metabolome, potentially playing a more dominant role than genetics (Loh et al., 2024). Therefore, based on the aforementioned research progress, we hypothesized that the GM plays a pivotal role in the mechanism by which CPF enhances seizure susceptibility, potentially via metabolic pathways in the MGBA. Our investigation is the first to delve into the GM and its relationship with seizure susceptibility induced by CPF using *16S* ribosomal RNA amplicon sequencing. Furthermore, we explored the potential therapeutic effects of transplanting fecal samples from healthy controls. And we aimed to identify the core genera through joint analysis with lithium pilocarpine-induced epilepsy. Finally, we aimed to unravel the intrinsic mechanisms through serum untargeted metabolomic analysis to shed light on how the GM influences epilepsy. Our study contributes to a better understanding of how the GM functions in epilepsy via MGBA. These insights may pave the way for developing novel therapeutic approaches from a new perspective.

## 2 Materials and methods

### 2.1 Animals and experimental design

Adult male Sprague–Dawley (SD) rats weighing 180 ± 20 g with specified pathogen free (SPF) grade were used in this experiment (Vital River Laboratory Animal Technology Co., China). After one week of quarantine, the animals were individually housed in sterilized acrylic cages. The temperature and humidity of the room were kept at 21 ± 1°C and 55%, respectively. The rats were maintained under a 12:12-h light/dark cycle (lights on at 6 am) with free access to standard rodent food (SPF Biotechnology Co., China) and pure water. All animal care and experiments were carried out in accordance with the National Institutes of Health guide for the care and use of Laboratory animals (NIH Publications No. 8023, revised 1978) and approved by the Institutional Ethical Committee for Animal Welfare of the Seventh Affiliated Hospital of Sun Yat-sen University and in strict accordance with the National Institutes of Health guidelines for animal use in research.

#### 2.1.1 Ciprofloxacin-induced seizure susceptibility in rats

**Figure 1:**
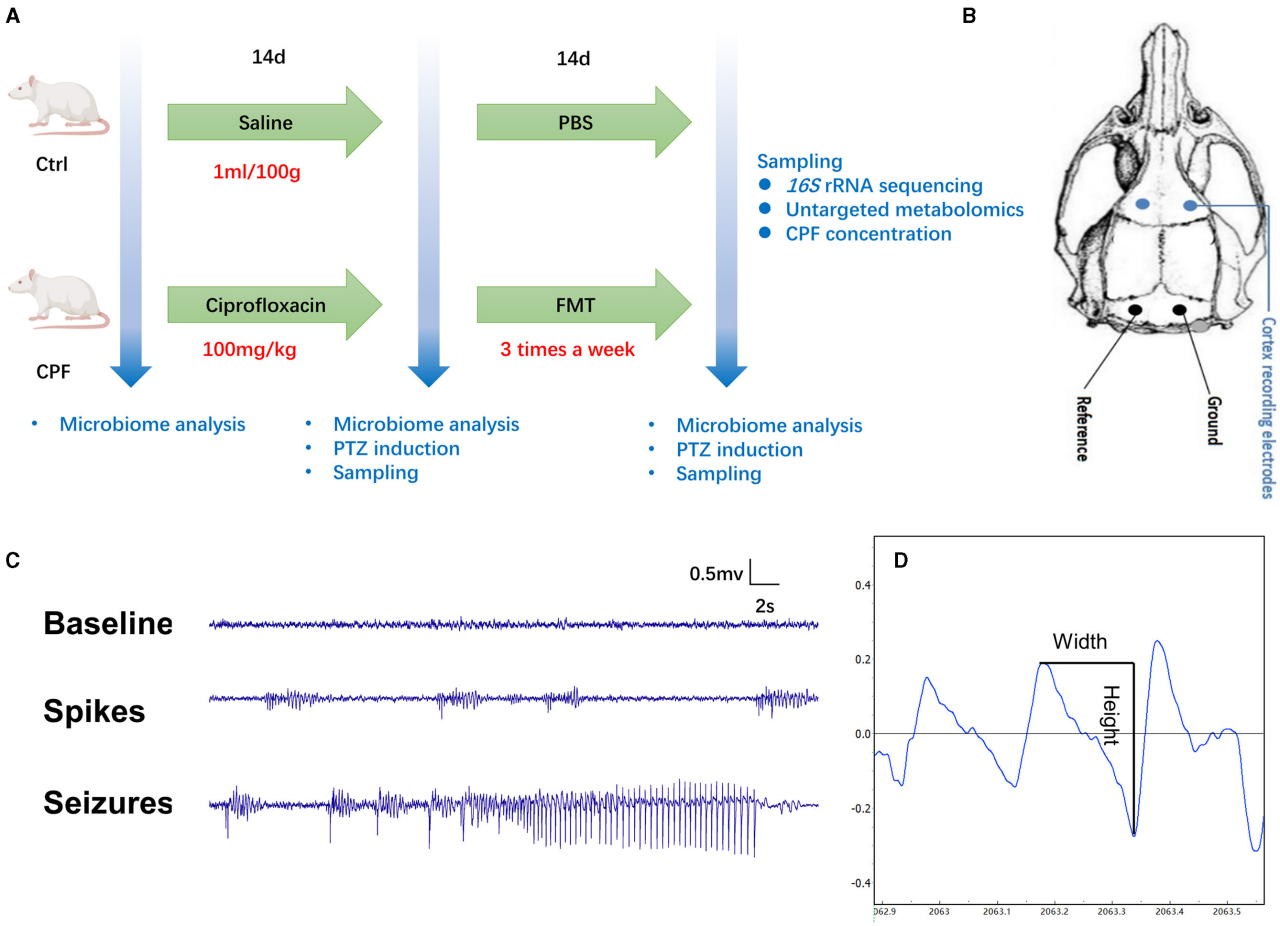
Flowchart depicting the study methodology and the assessment of seizure susceptibility. **(A)** The experimental design comprised rats receiving either CPF (*n* = 33) or NS (*n* = 30) gavage. On the 14th day, 19 rats in the CPF group (CPF-14 d) and 16 rats in the Ctrl group (CPF-14 d) underwent PTZ injection to assess seizure susceptibility and were subsequently sampled. The remaining rats continued FMT treatment (*n* = 14 in each group), and on the 28th day, 14 rats from both the CPF (CPF-FMT) and Ctrl groups (Ctrl-PBS) were tested for seizure susceptibility and sampled. **(B)** Epidural recording electrodes implantation. **(C)** An example of baseline, spikes and PTZ-induced seizure detected during the epilepsy susceptibility test with video monitoring. **(D)** The width and height in the spike mode pattern.

After 1 week adaption, all rats were randomly numbered and divided into two groups (Figure 1A).

Antibiotic Group (CPF *n* = 33): All the rats received ciprofloxacin monohydrochloride (TargetMol, USA) by gavage at a dose of 100 mg/kg body weight (dissolved in normal saline at 10 mg/mL) per day for 14 days. After CPF gavage, 19 rats were tested for seizure susceptibility (*n* = 6 at 45 mg/kg; *n* = 13 at 60 mg/kg), and the remaining 14 rats received fecal microbiota transplantation (FMT) three times a week for 14 days according to FMT procedure (Bokoliya et al., 2021).

Control Group (Ctrl *n* = 30): All rats were administered the same volume of normal saline (NS) by gavage for 14 days (*n* = 30). Subsequently, 16 rats were tested for seizure susceptibility (*n* = 6 at 45 mg/kg; *n* = 10 at 60 mg/kg). The remaining 14 rats received phosphate-buffered saline (PBS) at the same frequency as the CPF group, and served as fecal donors for FMT.

Seizure susceptibility testing was divided into two dose gradients (45 mg/kg and 60 mg/kg). The CPF dose used was calculated based on the daily dosage of the patient (1–1.5 g/d) and then adjusted according to the body surface area of the human and rat.

#### 2.1.2 Lithium pilocarpine-induced epilepsy rat model

Animals were divided into two groups: control (Normal) and epilepsy (Pilo). In the Pilo group, rats were injected intraperitoneally (i.p.) with lithium chloride (127.2 mg/kg). After 18 h, the rats were administered scopolamine (1 mg/kg, intraperitoneally). Thirty min later, pilocarpine hydrochloride (30 mg/kg, i. p.) was administered to induce status epilepticus. Seizure onset was identified visually and scored according to the Racine Scale (Racine, 1972). Rats that did not exhibit a score of 4 or above within 30 min of pilocarpine injection were repeatedly injected (10 mg/kg, i.p.) every 30 min until a scale 4 or above seizure was induced. We used 60 mg/kg as the maximum dose; otherwise, animals were not included in this study. Ninety min after the first grade 4 or higher seizure, diazepam (10 mg/kg, i.p.; Jinyao medicine, China) was administered to terminate the seizures. The control group (*n* = 3) was injected with 0.9% NS at each time point as the Pilo group. After 2 months, the rats were transferred to monitoring cages and monitored using an infrared night vision camera for 1 week. The monitored video results were analyzed in a double-blind manner by two senior investigators to identify seizures. Animals that experience spontaneous seizures are believed to develop epilepsy. Four rats were confirmed to have spontaneous seizures. Finally, fecal and serum samples from the two groups were collected for *16S* rRNA sequencing and LC-MS/MS analysis.

### 2.2 Electrode implantation

After CPF or FMT gavage, the animals were surgically implanted with epidural recording electrodes for seizure susceptibility testing and video-electroencephalogram (VEEG) monitoring. Rats were anesthetized with isoflurane (5% anesthesia induction) for 2 min, and anesthesia was maintained at an isoflurane concentration of 2%. After anesthesia, the rats were mounted on a stereotaxic apparatus (RWD, Shenzhen). According to the brain coordinates identified in the rat stereotaxic atlas by Paxinos and Watson (2007), four burr holes were drilled through the skull. For electroencephalogram (EEG) recording, two stainless skull screw electrodes (Selectaplus, Dentsply, DeTrey GmbH, Dreieich, Germany; 1.0 mm in diameter) were advanced into the bilateral motor cortex (anteroposterior + 3.0 mm, mediolateral ± 2.5 mm), and two into the skull bilaterally over the cerebellum to serve as a reference and ground electrodes (Figure 1B). The reference and ground skull screw electrodes were placed extra-axially, overlying the cerebellum. All electrodes were secured using dental cement.

### 2.3 VEEG recording and seizure susceptibility test

Two days after surgery, the rats were placed in custom-made transparent cages and allowed to move freely (one rat/cage). EEG signals were recorded and digitized using a PowerLab8/35 system (Analog-to-Digital Converter Instruments, Colorado Springs, CO, USA) at a sampling rate of 2 kHz using synchronized video recording. EEG signals were digitally filtered (high-pass at 0.5 Hz, low-pass at 70 Hz, 50 Hz notch filter) and examined for the presence of spikes. The behaviors of the animals were recorded using a camera. We first recorded 10-min baseline EEG signals. After baseline recording, all animals were injected intraperitoneally with a single dose of pentylenetetrazole (PTZ) to induce acute seizures, which was dissolved in NS (18 mg/mL or 24 mg/mL). VEEG was recorded for 90 min (Figure 1C). The onset and termination of a seizure are readily recognizable as abrupt changes in EEG frequency, amplitude, and epileptic behavior. The seizure behavioral scale criteria based on the Racine scale were as follows: Score 0: no response; Score 1: staring and mouth clonus; Score 2: head nodding; Score 3: unilateral forelimb clonus; Score 4: rearing and bilateral forelimb clonus; and Score 5: rearing and losing the balance (Racine, 1972). The number of seizures, average and accumulated duration of seizures, latency to the first spike cluster and first seizure, total power change (0–500 Hz), and number of single spikes were calculated as indicator parameters for epilepsy susceptibility. A Fast Fourier Transformation was used to calculate the total power in the 0–500 Hz frequency band. EEG spikes were defined as high-voltage (>4 SD above background) positive or negative single deflections that lasted < 50 ms in 90-min recording screened using the above-mentioned software PowerLab8/35 system (Figure 1D) (Mengoni et al., 2021). Each putative spike detected and electrographic seizures were verified in a blinded manner by two independent observers who were not aware of the treatment of the animals.

### 2.4 Weight record and samples collection

Body weight was recorded every 3d throughout the duration of gavage to adjust the volume of CPF or fecal slurry. Fecal samples were collected at specific time points during the study: before CPF treatment initiation (day 0), 14 d after CPF/NS gavage, and 2 weeks after FMT. The collection time was standardized between 9:00 and 11:00 AM to minimize variations due to circadian effects. Two to three fecal boluses were collected aseptically from each animal. To ensure that the collected feces were not polluted by the environment, the samples were directly deposited into 1.5 mL sterile Eppendorf (EP) microtubes. Then, the samples were promptly transferred to a freezer set at −80°C for long-term storage until further analysis. The day after the susceptibility test, the rats were euthanized, and the brain tissue was quickly removed and frozen at −80°C for subsequent experiments.

### 2.5 Normal fecal microbiota transplantation

For normal fecal suspension preparation, fresh fecal samples were obtained from control rats. Approximately 1 g of fecal samples (equivalent to approximately five fresh fecal samples) was immediately steeped and homogenized in 5 mL of PBS. The mixture was then centrifuged at 2,500 rpm (500 × g) for 10 min at 4°C to pellet insolubilized material. The supernatant underwent further processing by passing it through a nylon filter with a pore size of 40 μm to effectively eliminate particulate and fibrous matter, generating the microbiome suspension (Gheorghe et al., 2021). After the 2-day antibiotics washout period, each rat in the CPF group received 1 mL/100 g body weight fecal slurry from the control group via oral gavage three times a week for 2 weeks.

### 2.6 Fecal DNA extraction, *16S* rRNA sequencing and bioinformatics analysis

Fecal DNA was extracted using the ALFA-SEQ Advanced Stool DNA Kit (Findrop, Guangzhou, China) according to the manufacturer's instructions. The concentration and purity were assessed using a NanoDrop One (Thermo Fisher Scientific, MA, USA). The V3-V4 hypervariable region of the *16S* ribosomal RNA genes was amplified using specific primer carrying Illumina overhang adapter sequences (forward: 515F: 5′-GTGCCAGCMGCCGCGGTAA-3′; reverse 806R: 5′-GGACTACHVGGGTWTCTAAT-3′) with a 12 bp barcode. The primers were synthesized by Invitrogen (Carlsbad, CA, USA). The length and concentration of the PCR products were determined using 1% agarose gel electrophoresis. Samples with a bright strip between 290 and 310 bp were used for further experiments. The library was sequenced on an Illumina Nova 6000 platform and 250 bp paired-end reads were generated (Guangdong Magigene Biotechnology Co. Ltd. Guangzhou, China). Paired-end clean reads were merged using usearch fastq_mergepairs. Fastp (version 0.14.1, https://github.com/OpenGene/fastp) was used to control the quality of the raw data using a sliding window (-W4-M20) to obtain paired-end clean tags. The raw sequences were processed using the UPARSE pipeline in R to generate operational taxonomic units (OTUs), Taxonomy was assigned using Greengenes (http://greengenes.lbl.gov/) database, and taxonomic information was annotated using usearch-sintax (by setting the confidence threshold to default to ≥0.8).

Alpha- and beta-diversity calculations were performed using the QIIME2 and R software (v3.6.1). The Chao1 and Shannon_2 indices in our samples were calculated using alpha_div (V10, http://www.drive5.com/usearch/). Principal coordinate analysis (PCoA) was based on the unweighted UniFrac distance, and the *p*-value of the analysis of similarities (ANOSIM) was obtained using a permutation test. We then conducted a linear discriminant analysis effect size (LEfSe) to determine the differential taxa based on the homogeneous OTUs table. Phylogenetic investigation of communities by reconstruction of unobserved state 2 (PICRUST2, https://huttenhower.sph.harvard.edu/picrust/) was used to predict the function and metabolic enzymes of *16S* rDNA.

### 2.7 Serum untargeted metabolomic analysis

Blood samples were collected from SD rats (Ctrl and CPF) at three specific time point (0, 14, and 28 d), and from rats in the Normal and Pilo groups after model validation. For serum extraction, blood samples collected from the tail and portal veins were stored at 22 ± 2°C for 60 min, centrifuged at 3,500 rpm for 15 min at 25°C, and the supernatant was stored at −80°C. Fifty microliters of rat serum were transferred to an EP tube. After the addition of 200 μL of extract solution (acetonitrile: methanol = 1: 1, containing isotopically-labeled internal standard mixture), the samples were vortexed for 30 s, sonicated for 10 min in ice-water bath, and incubated for 1 h at −40°C to precipitate proteins and centrifuged at 12,000 rpm (RCF = 13,800 × g, R = 8.6 cm) at 4°C for 15 min. The resulting supernatants were transferred to fresh glass vials for further analysis. A quality control sample was prepared by mixing equal aliquots of the supernatant from each sample. Untargeted metabolite data analysis was performed LC-MS/MS analyses were performed using an UHPLC system (Vanquish, Thermo Fisher Scientific) with a UPLC BEH Amide column (2.1 mm × 100 mm, 1.7 μm) coupled to Q Exactive HFX mass spectrometer (Orbitrap MS, Thermo Fisher Scientific).

### 2.8 Determination of ciprofloxacin concentration in rat cortex

Brain tissue samples were thawed at 4°C and 30 mg of tissue was added to 400 μL 75% methanol-water, swirled for 60 s, fully ground with small steel balls, placed in liquid nitrogen for 1 min, thawed at room temperature, and ultrasound on ice for 10 min (repeated three times). Finally, the mixture was centrifuged at 17,000 × g at 4°C for 15 min, and filtration was done using a 0.22-μm organic membrane filter head.

The internal standard is diluted with 50% methanol-water to achieve a specific concentration of the internal standard diluent. A diluent containing the internal standard was used to dilute the mixed standard and sample 1:1 by volume. The final internal standard concentration was 100 ng/mL. Parameters: Column temperature: 40°C, loading volume: 1 μL, positive ion mode A: 0.1% formic acid aqueous solution. B: Acetonitrile. Mass spectrum conditions: based on the Multiple Reaction Monitoring mode, the AB4500 mass spectrometer collected the primary and secondary mass spectrum data in negative mode. Electrospray Ionization ion source parameters are set as follows: Gas temp: 500°C, Curtain gas: 25 Psi, Collision gas: 10 Psi, ionspray voltage: 4500 V, atomization temperature: 500°C.

### 2.9 Statistical analysis

For two-group comparisons, the unpaired Student's *t*-test or Mann–Whitney U test was performed. The Cox proportional hazards model was used to compare seizure onset rates. GraphPad Prism 8.02 and SPSS 25.0 were used for statistical analysis and figure production. Statistical significance was set at *p* < 0.05.

## 3 Results

### 3.1 Modulation of rat seizure susceptibility via CPF-induced alterations in GM

#### 3.1.1 CPF gavage increases the seizure susceptibility in SD rats

**Figure 2:**
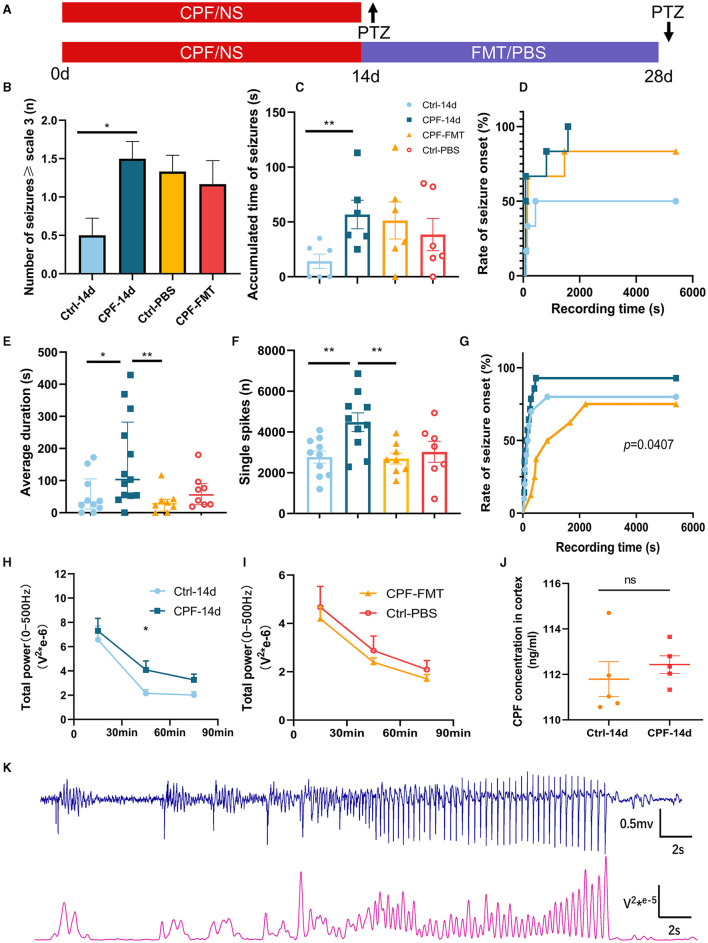
Modulation of seizure susceptibility via CPF-induced alterations in GM. **(A)** Schematic diagram of experimental process. **(B)** The number of seizures ≥ scale 3 (PTZ 45 mg/kg). **(C)** The accumulated time of seizures (PTZ 45 mg/kg). **(D)** The rate of seizure onset (PTZ 45 mg/kg). **(E)** The average duration of each seizure (PTZ 60 mg/kg). **(F)** The number of single spikes during recording 90 min (PTZ 60 mg/kg). **(G)** The rate of seizure onset (PTZ 60 mg/kg). **(H)** Total power of 0–500 Hz after 14-day CPF gavage (PTZ 60 mg/kg). **(I)** Total power of 0–500 Hz after FMT (PTZ 60 mg/kg). **(J)** CPF concentration in cortex after CPF gavage (*n* = 5 in each group). **(K)** A part of the correspondence between EEG and 0–500 Hz power curves. Statistics: normally distributed data are given as the mean ± SEM, others are presented as median with interquartile range. *p < 0.05, **p < 0.01.

To determine whether a daily dose of CPF via gavage could enhance seizure susceptibility in SD rats, we induced acute seizure attacks by administering a single i.p. injection of PTZ after 14-day period of CPF or NS gavage (Figure 2A).

Initially, we evaluated seizure susceptibility using 45 mg/kg (18 mg/ml) PTZ (*n* = 6 each in CPF and Ctrl groups). A significant increase in the number of seizures ≥ scale 3 was observed in the CPF-14 d group compared with that in the Ctrl-14d (*p* < 0.05, Figure 2B). Furthermore, there was a substantial increase in the accumulated time of seizures (*p* < 0.01, Figure 2C). To confirm these results, we conducted a seizure susceptibility test with 60 mg/kg (24 mg/mL) PTZ (*n* = 13 in CPF, *n* = 10 in Ctrl). While the number of seizures ≥ scale 3 did not exhibit a significant difference, the CPF-14 d demonstrated a significant augmentation in the average duration of each seizure compared with that in Ctrl-14d (*p* < 0.05, Figure 2E). In addition, the occurrence of single spikes during the 90-min recording significantly increased (*p* < 0.01, Figure 2F). The power can also reflect the EEG discharge (Figure 2K), and the total power in the 0–500 Hz range during the 30 to 60-min interval demonstrated a significant increase in the CPF-14 d, indicating a prolonged period of excitation following CPF gavage (*p* < 0.05, Figure 2H). The death rate during seizures in the CPF-14 d group was 23.1%, whereas none of the rats in the control group died during seizures.

To avoid CPF from entering the brain and causing seizures, we measured CPF concentrations in the cortex. To eliminate this possibility, our results showed that there was no significant difference in CPF concentration in the cortex between the CPF and control group after the 14-day gavage and washout period (Figure 2J).

#### 3.1.2 FMT can reduced seizure susceptibility caused by CPF

To examine whether restoring the GM could reduce the increased seizure susceptibility caused by CPF, we also detected seizure susceptibility after FMT between the CPF-FMT and Ctrl-PBS groups.

After 45 mg/kg PTZ injection (*n* = 6), no significant differences were observed in the number of seizures and accumulative seizure duration following FMT treatment compared to Ctrl-PBS group (Figures 2B, C). The seizure onset rates were 50%, 100%, and 83% in Ctrl-14 d, CPF-14 d, and CPF-FMT, respectively (Figure 2D).

Next, we used 60 mg/kg PTZ to detect seizure susceptibility. FMT treatment resulted in a significant decrease in the average duration of seizures (CPF-14 d vs. CPF-FMT, *p* < 0.01, Figure 2E) and the occurrence of single spikes decreased (CPF-14 d vs. CPF-FMT, *p* < 0.01, Figure 2F), approaching the levels seen in Ctrl-PBS. There was no significant difference in the total power between the two groups in the total power in 0–500 Hz range after FMT (Figure 2I). Moreover, the percentage of seizure onset between CPF-14 d, Ctrl-14 d, and CPF-FMT also exhibited a trend similar to that of 45 mg/kg PTZ (*p* = 0.0407, Figure 2G).

### 3.2 Gut dysbiosis caused by CPF gavage is similar to that in the lithium pilocarpine-induced epilepsy rat model and can be relieved by FMT

#### 3.2.1 GM diversity in the two rat models

**Figure 3:**
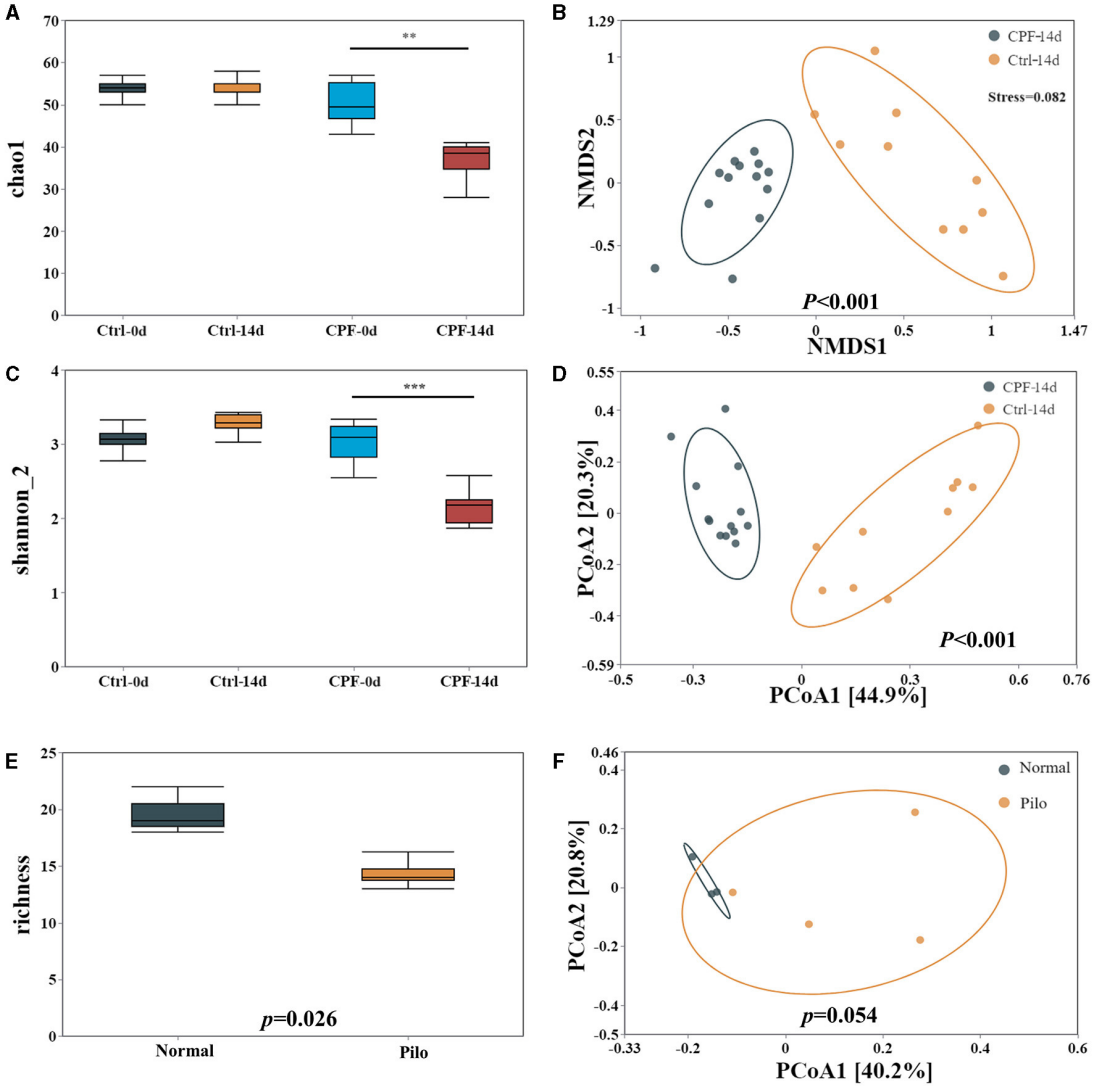
Alterations in GM diversity in CPF-induced seizure susceptibility and lithium pilocarpine-induced epilepsy rat models. **(A)** Alpha diversity in the model of CPF-induced seizure susceptibility in Chao1. **(B)** Beta diversity in the model of CPF-induced seizure susceptibility in NMDS2. **(C)** Alpha diversity in the model of CPF-induced seizure susceptibility Shannon_2. **(D)** Beta diversity in the model of CPF-induced seizure susceptibility in PCoA. **(E)** Alpha diversity in the model of lithium pilocarpine-induced epilepsy. **(F)** Beta diversity in the model of lithium pilocarpine-induced epilepsy. Statistics: Student *t* test was used for two-group comparison and Kruskal-Wallis test was used for multiple groups. **p < 0.01, ***p < 0.001.

Results from *16S* rRNA sequencing of the GM in different groups showed that CPF gavage markedly disturbed GM diversity, exhibiting a pattern similar to that of the lithium pilocarpine-induced epilepsy rat model (Figure 3).

First, in the CPF-induced seizure susceptibility model, OTUs decreased after CPF treatment. Following Illumina sequencing and application of the 97% similarity threshold, the detected OTUs were similar between the rats in the Ctrl and CPF groups at the start of the experiment, with similar OTUs numbers (CPF-0 d = 509, Ctrl-0 d = 514). However, the number of OTUs significantly decreased to 374 in the CPF-14 d group, while the Ctrl-14 d group contained 613 OTUs.

Second, alpha diversity analysis revealed a reduction in richness and diversity during CPF gavage. As measured using Chao1 and Shannon_2, alpha-diversity significantly decreased in CPF-14 d group compared with that in the Ctrl-14 d group (*p* < 0.01 Chao1, *p* < 0.001, Shannon_2, Figures 3A, C). Moreover, the PCoA analysis of the beta-diversity of the GM quantified by the Bray–Cuitis distance indicated a clear distinction between CPF-14 d and Ctrl-14 d groups (Anosim R = 0.946, *p* < 0.001) shown in Non-metric Multidimensional Scaling (NMDS) (Figure 3B, stress = 0.082) and PCoA (Figure 3D).

Notably, we observed a similar trend in GM diversity in a lithium pilocarpine-induced epilepsy model. Alpha diversity was also significantly decreased in the Pilo group in terms of richness (*p* = 0.026; Figure 3E). PCoA for beta diversity revealed a distinction between the Pilo and Normal groups, although the difference was not statistically significant (*p* = 0.054, Figure 3F).

#### 3.2.2 Composition and differential microbiota in the two rat models

Having identified clear changes in the GM diversity, we aimed to obtain more detailed information, including microbial community composition and species differences. We considered all detailed data regarding different taxa at the phylum and genus levels, with a relative abundance of over 0.01% and the top 15 genera.

**Figure 4:**
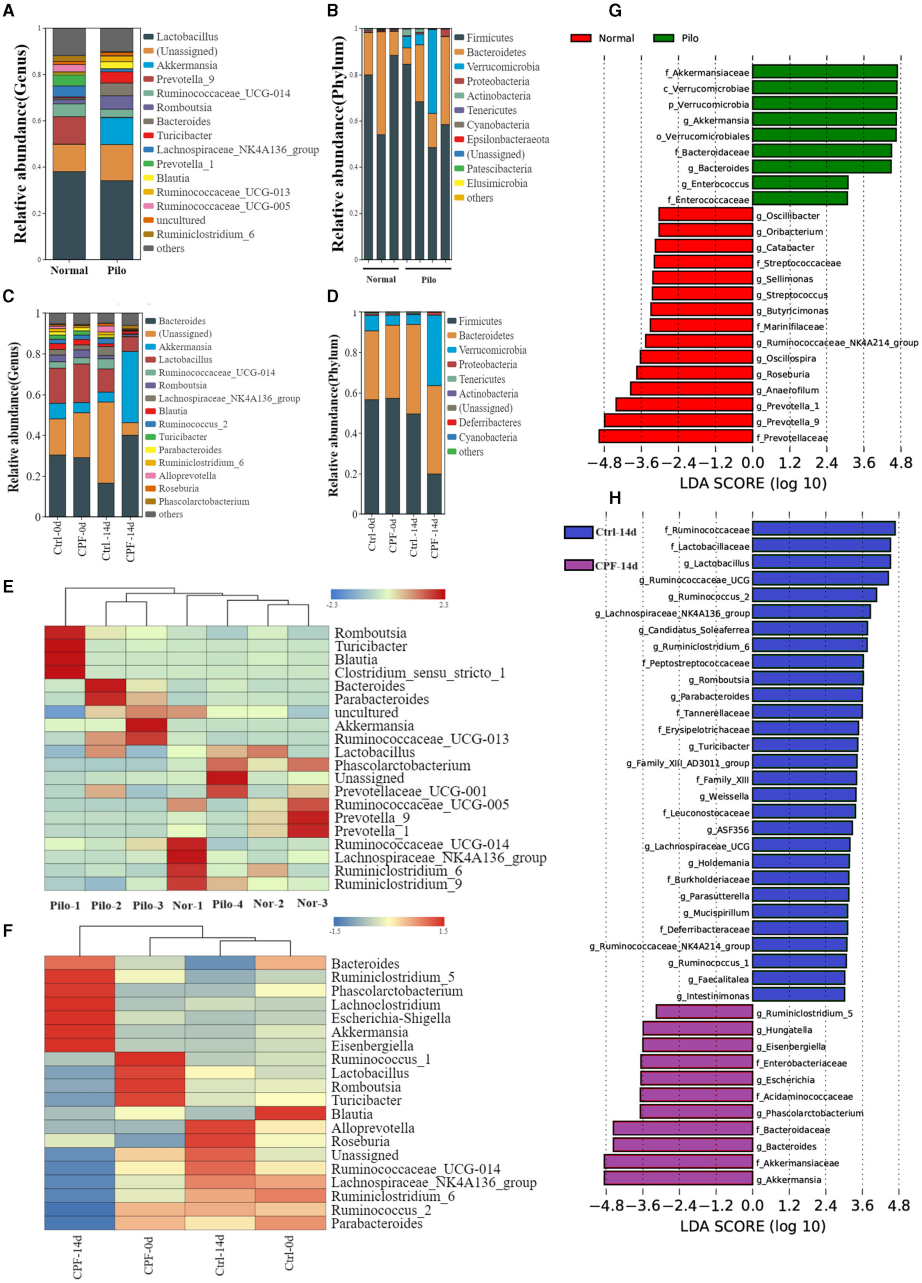
Analysis of the differential GM in CPF-induced seizure susceptibility and lithium pilocarpine-induced epilepsy rat models. **(A, B)** Microbial composition at the genus and phylum levels between the Pilo and Normal group (top 15 microbiota with relative abundance >0.01%). **(C, D)** Microbial composition at the genus and phylum levels between the CPF and Ctrl group at different timepoints (top 15 microbiota with relative abundance >0.01%). **(E)** Heatmap of relative abundance at the genus level between Pilo and Normal group. **(F)** Heatmap of relative abundance at the genus level between CPF and Ctrl group. **(G)** Discriminant taxa between Pilo and Normal group ranked by their linear discriminant analysis (LDA) effect size (LEfSe) of microbiota, LDA score >3. **(H)** Discriminant taxa between CPF and Ctrl group at 14^th^ day (CPF-14 d vs. Ctrl-14 d) ranked by LEfSe, LDA score >3.

In the CPF-induced seizure susceptibility model, Firmicutes and Bacteroidetes were the predominant bacterial phyla. Prior to gavage, the percentages of Firmicutes (65.0% in Ctrl-0 d, 57.3% in CPF-0 d) and Bacteroidetes (30.2% in Ctrl-0 d, 34.4% in CPF-0 d) were similar between the two groups. However, Firmicutes decreased to 21.9% after CPF administration. Notably, the relative abundance of Verrucomicrobia, which ranked third, increased substantially after CPF gavage, increasing from 7.1% to 38.1% (Figure 4D). At the genus level, *Lactobacillus, Ruminococcaceae, Parabacteroides, Ruminococcaceae_NK4A214_group*, and *Butyricicoccus* exhibited reduced relative abundance following CPF gavage. Conversely, certain bacterial taxa experienced a relative increase, including *Bacteroides, Akkermansia, Eisenbergiella*, and *Phascolarctobacterium* (Figure 4C). Furthermore, research on presumed distinctive microbial differences between the two groups was performed using LEfSe analysis. As illustrated in Figure 4H, *Akkermansia, Bacteroides, Phascolarctobacterium, Escherichia, Hungatella*, and *Ruminiclostridium_5* were identified as characteristic bacteria in CPF-14 d. In contrast, *Lactobacillus, Ruminococcus_2, Romboutsia, Parabacteroides*, etc., were found to be the characteristic bacteria in Ctrl-14 d. A heat map of the relative abundances is shown in Figure 4F.

In the lithium pilocarpine-induced epilepsy model, the GM composition was entirely different from that of the normal group. Firmicutes (74.5% in Normal and 65.1% in Pilo) and Bacteroidetes (24.1% in Normal and 20.9% in Pilo) were the two most abundant phyla. Similar to the increased relative abundance of CPF-induced seizure susceptibility, the relative abundance of Verrucomicrobia was much higher in the Pilo group (11.6% in Pilo and 0.04% in Normal groups; Figure 4B). At the genus level, *Lactobacillus, Prevotella-9*, and *Lachnospiraceae_NK4A136_group* exhibited reduced relative abundance in the Pilo group. However, the abundances of *Akkermansia, Romboutsia*, and *Bacteroides* increased (Figure 4A). LEfSe analysis revealed that *Akkermansia, Bacteroides*, and *Enterococcus* were the characteristic bacteria in Pilo. In contrast, *Prevotella, Anaerofilum, Roseburia, Oscillospira, Butyricicoccus*, and *Ruminococcaceae_NK4A214_group* were the characteristic bacteria under normal conditions (Figure 4G). A heat map of the relative abundances is shown in Figure 4E. Most importantly, we found some commonalities in GM diversity and compositional changes in both models.

#### 3.2.3 FMT treatment reconstituted GM dysbiosis and reduction after CPF gavage

**Figure 5:**
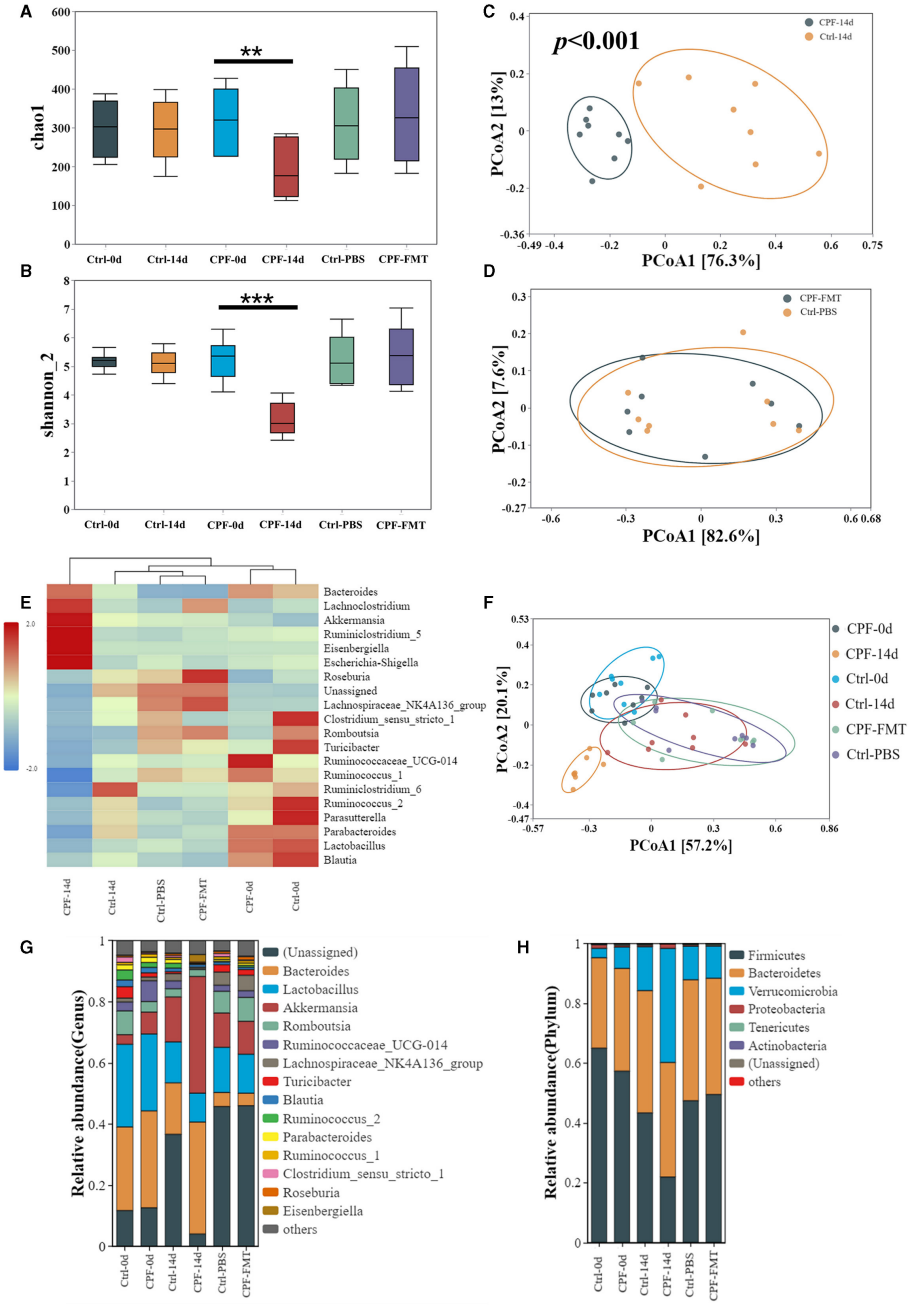
FMT treatment reconstituted GM dysbiosis after CPF gavage. **(A, B)** Alpha diversity of the GM between groups at different timepoints (0 d, 14 d, 28 d) in Chao1 and Shannon_2. **(C)** Beta diversity of the GM beween CPF-14 d and Ctrl-14 d in PCoA (Anosim R = 0.946, *p* < 0.001). **(D)** Beta diversity of the GM beween CPF-FMT and Ctrl-PBS in PCoA. **(E)** Heatmap of relative abundance at the genus level at different timepoints (0 d, 14 d, 28 d). **(F)** Beta diversity of the GM beween CPF and Ctrl group at different timepoints (0 d, 14 d, 28 d). **(G, H)** Microbial composition at the phylum and genus levels between the CPF and Ctrl groups at different timepoints (0 d, 14 d, 28 d) (top 15 microbiota with relative abundance >0.01%). Statistics: Student *t* test was used for two-group comparison and Kruskal-Wallis test was used for multiple groups. **p < 0.01, ***p < 0.001.

Following FMT, we performed *16S* rRNA sequencing and subsequent analysis to verify the restoration of the GM profile. Post-FMT, the number of OTUs increased to 699 in the CPF-FMT group compared with 609 in the Ctrl-PBS group. Furthermore, the alpha-diversity metrics, as indicated by the Chao1 and Shannon_2 indices, reverted to levels comparable to those of the Ctrl-PBS (Figures 5A, B). Beta-diversity analysis revealed an overlap between the CPF-FMT and Ctrl-PBS samples (Figure 5D), in contrast to the distinct separation observed between the CPF-14 d and Ctrl-14 d (Figure 5C). However, while recovery trended toward Ctrl-PBS, it did not completely overlap with Ctrl-0 d and Ctrl-14 d (Figure 5F), underscoring the dynamic shifts in the GM throughout growth and development. Consistent alterations were also evident in the phylum and genus compositions (Figures 5G, H), along with their relative abundances (Figure 5E), showing that FMT helped gut dysbiosis recover to that of the Ctrl group.

#### 3.2.4 The common alteration of genera and the change of seizure susceptibility

There were some similar changes in the GM of the CPF-induced seizure susceptibility and lithium pilocarpine-induced epilepsy models. To explore the core GM that might have shared potential to influence susceptibility to epilepsy, we conducted a comparative and correlated analysis of the GM profiles between CPF-induced seizure susceptibility and lithium pilocarpine-induced epilepsy rat models.

**Figure 6:**
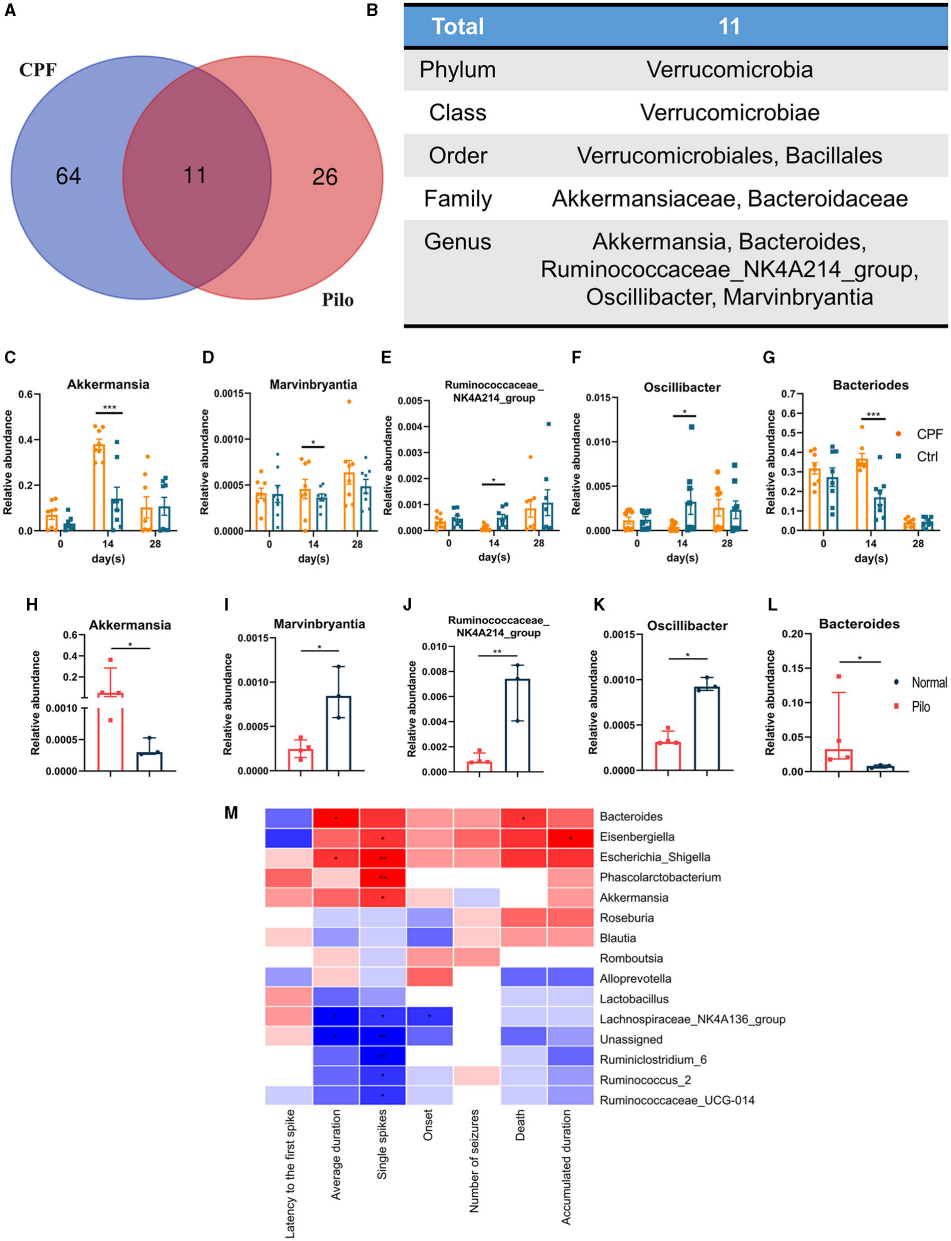
Common alterations observed in some genera in CPF-induced seizure susceptibility and lithium pilocarpine-induced epilepsy rat models. **(A)** Venn diagram of GM of CPF-induced seizure susceptibility and lithium pilocarpine-induced rat models. **(B)** The total 11 differential GM in all classification levels. **(C–G)** Relative abundances of five significantly altered bacterial genera in CPF compared to Ctrl. **(H–L)** Relative abundances of five significantly altered bacterial genera in Pilo compared to Normal. **(M)** Correlation heatmap of seizure susceptibility and GM abundances. Statistics: normally distributed data are given as the mean ± SEM, others are presented as median with interquartile range. *p < 0.05, **p < 0.01, ***p < 0.001.

The results of LEfSe analysis in both models were compared to identify common changes in the GM. In total, we observed 11 common taxa that exhibited the same significant change in direction in both models (Figures 6A, B). At the genus level, *Akkermansia* and *Bacteroides* increased in both models, while *Marvinbryantia, Oscillibacter*, and *Ruminococcaceae_NK4A214_group* decreased. The relative abundances of the above five genera after CPF and FMT treatments are shown in Figures 6C–G, and the relative abundances in Pilo compared to Normal are shown in Figures 6H–L. Notably, we discovered that *Akkermansia* increased in both the CPF-14 d and Pilo groups, with a noticeable increase spanning the entire phylum. From the phylum to genus level, there was a notable increase in Verrucomicrobia, *Verrucomicrobiae, Verrucomicrobiales, Akkermansiaceae*, and *Akkermansia*. Correlation analysis revealed that *Bacteroides, Eisenbergiella, Escherichia_Shigella, Phascolarctobacterium*, and *Akkermansia* were positively associated with heightened seizure susceptibility. In contrast, *Lachnospiraceae_NK4A136_group, Ruminiclostridium_6, Ruminococcus_2*, and *Ruminococcaceae_UCG-014* showed negative correlations (Figure 6M).

### 3.3 Untargeted metabolomics profile changed in serum after CPF gavage

**Figure 7:**
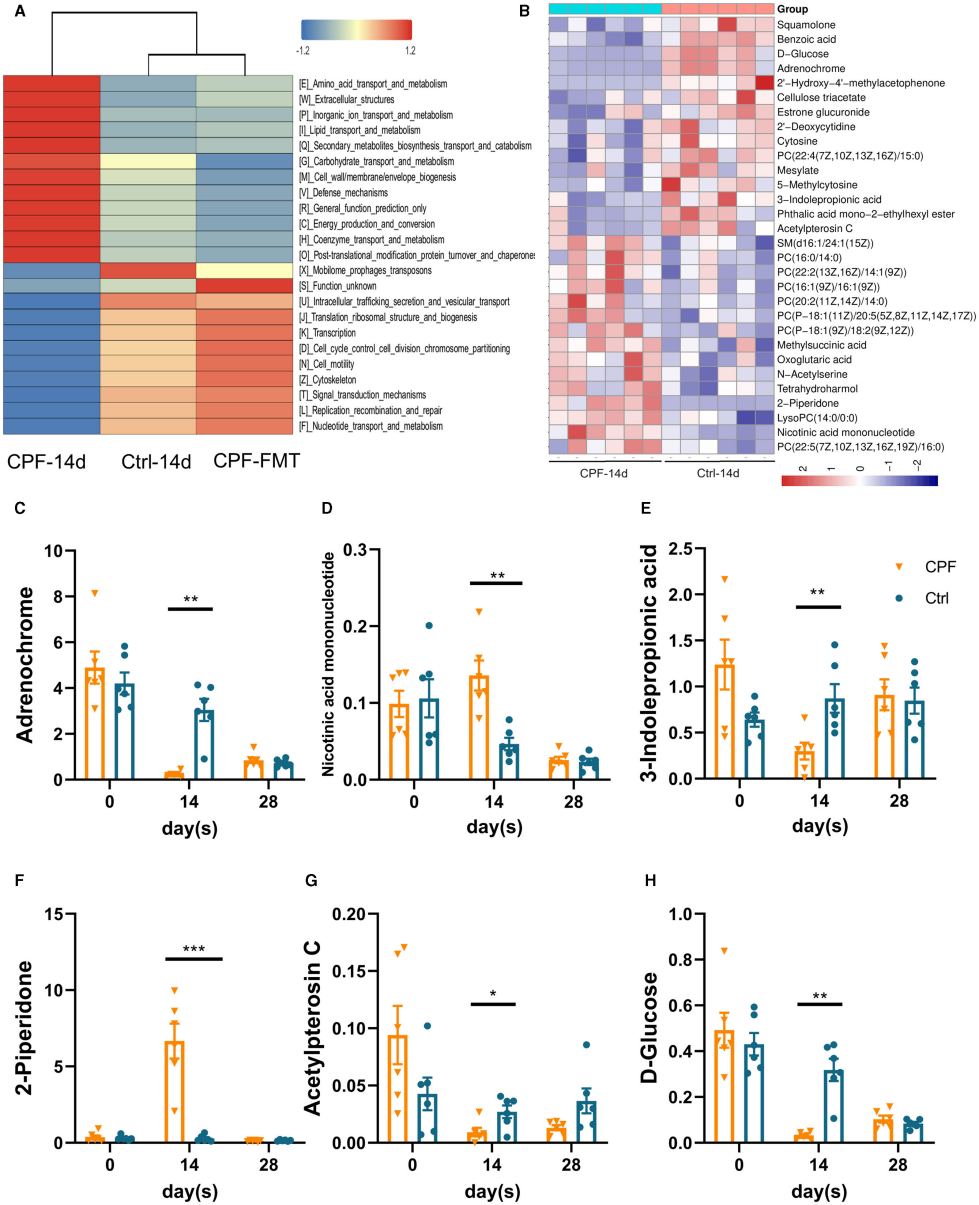
Untargeted metabolomics shifted in the serum after CPF gavage and FMT. **(A)** Comparison of PICRUSt-based metagenomics prediction among Ctrl-14 d, CPF-14 d and CPF-FMT. **(B)** Heatmap of hierarchical clustering analysis between CPF-14 d and Ctrl-14 d of the serum untargeted metabolism. **(C–H)** The significant different metabolites affected by CPF and FMT treatment (3-indolepropionic acid, acetylpterosin C, 2-piperidone, D-glucose, Nicotinic acid mononucleotide, and Adrenochrome). Statistics: normally distributed data are given as the mean ± SEM, *p < 0.05, **p < 0.01, ***p < 0.001.

To further investigate how GM influences epilepsy and its susceptibility mechanisms, we explored how GM affects host metabolism in serum. Based on the differential GM, the analysis of microbial functional prediction using PICRUSt2 revealed that the metabolic patterns in serum were similar between Ctrl-14 d and CPF-FMT but dissimilar to CPF-14 d in KEGG Orthology. Some processes, including amino acid transport and metabolism, extracellular structure, and lipid metabolism, exhibited similarities (Figure 7A). The heatmap shows the correlation between different metabolites and groups (Figure 7B). Among the discriminant metabolites in CPF-14 d, we identified a decrease in Adrenochrome, Acetylpterosin C, and 3-indolepropionic acid (IPA) compared to those in Ctrl-14 d. D-glucose, the main energy source for the brain, also decreased. However, 2-piperidone and nicotinic acid mononucleotides increased (variable importance of projection >1, *p* < 0.05, Figures 7C–H).

### 3.4 Dynamic changes in IPA serum levels in CPF-induced seizure susceptibility and lithium pilocarpine-induced epilepsy rat models

**Figure 8:**
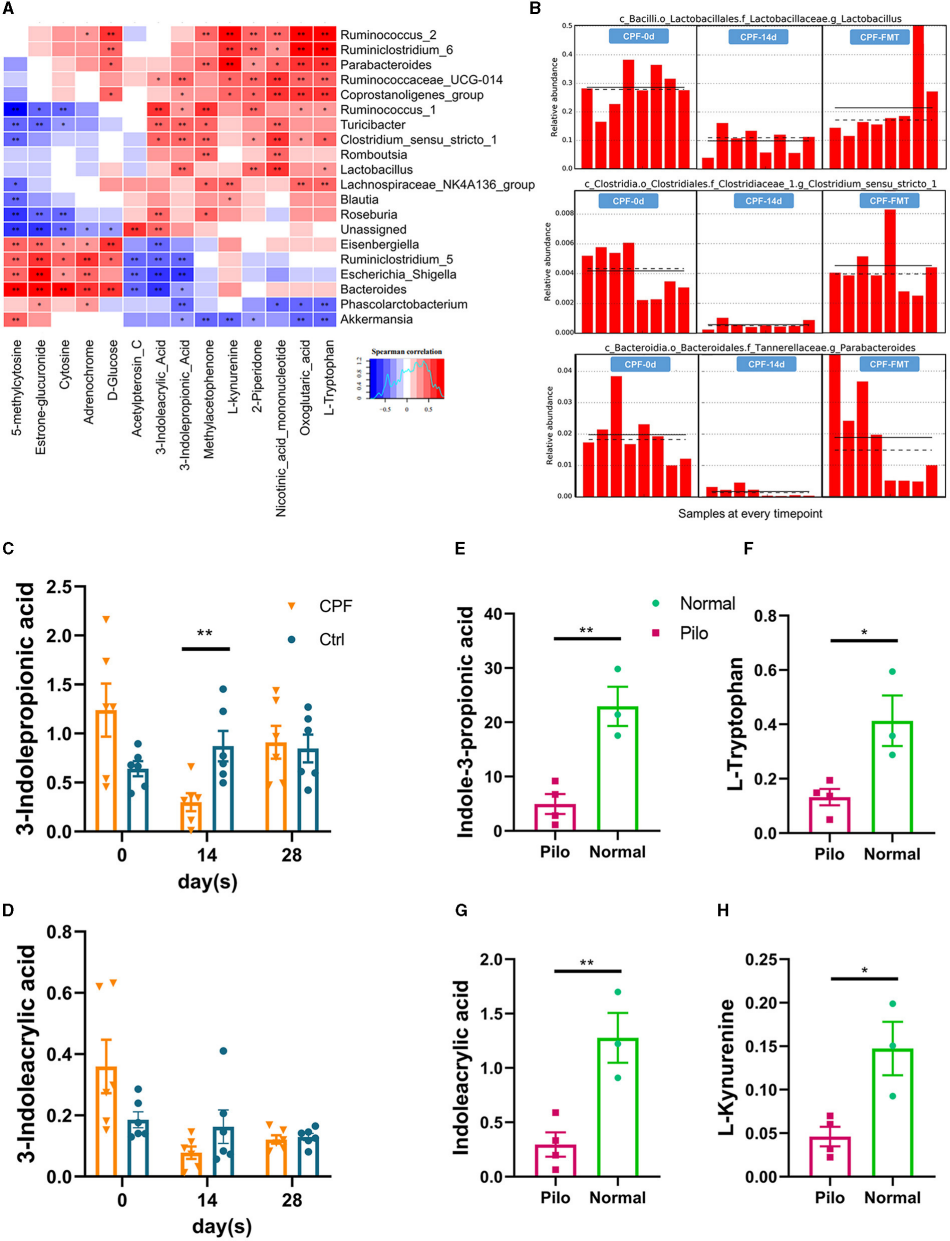
Dynamic modulation of 3-indolepropionic acid in CPF-induced seizure susceptibility and lithium pilocarpine-induced epilepsy rat models. **(A)** Microbial community and untargeted serum metabolomics correlation heatmap analysis. **(B)** The relative abundances of tryptophan-indole metabolism related GM in different timepoints by lefSE analysis (*Lactobacillus p* = 0.00041, *Parabacteroides p* = 0.00043, and *Clostridiaceae_1 p* = 0.00046). **(C, D)** Changes of serum metabolites related to tryptophan-indole metabolism in CPF group. **(E–H)** Changes of serum metabolites related to tryptophan-indole metabolism in lithium pilocarpine-induced epilepsy model. Statistics: normally distributed data are given as the mean ± SEM, *p < 0.05, **p < 0.01.

In a cross-analysis of the similarities and differences in serum metabolomics between the two rat models, we observed a consistent decrease in the common metabolite, IPA, a key metabolic product of tryptophan. The heatmap illustrated clear associations between certain GM taxa and altered metabolites (Figure 8A). The GM taxa that were significantly related to these changes included *Parabacteroides, Ruminococcus_1, Ruminococcus_2, Ruminiclostridium_6, Eisenbergiella, Escherichia_shigella, Bacteroides, Phascolarctobacterium*, and *Akkermansia*. We found that some indole-associated bacteria, including *Lactobacillus, Parabacteroides*, and *Clostridiaceae_1*, were consistent with the changes in IPA during CPF gavage and FMT (Figure 8B). In the CPF group, there was a significant decrease in IPA (*p* < 0.01). Although 3-indoleacrylic acid levels also decreased, this decrease did not reach statistical significance (Figures 8C, D). Notably, the levels of corresponding indole derivatives showed a more pronounced decrease in the Pilo group, along with reduction in the levels of other products associated with tryptophan, including L-tryptophan, and L-kynurenine (Figures 8E–H).

## 4 Discussion

In this study, we demonstrated for the first time that the therapeutic dose of oral CPF increases susceptibility to epilepsy by directly altering the GM and its metabolites, without increasing CPF concentrations in the brain tissue. Moreover, in a more detailed analysis, we observed the diversity, composition and identical changes in some core genera in CPF-induced seizure susceptibility and lithium pilocarpine-induced epilepsy rat models. These changes may play a crucial role in epilepsy. Finally, we identified one of the metabolites of the tryptophan-indole metabolic process, IPA, as potentially significant in the pathological process of epilepsy. This study provides valuable insights into the intricate interplay between the GM and epilepsy, opening avenues for refined treatment strategies for epilepsy.

Numerous clinical studies have demonstrated that some antibiotics can induce epilepsy or increase susceptibility to epilepsy, including β-lactam, fluoroquinolone families, fourth-generation cephalosporins, and carbapenems (Wanleenuwat et al., 2020). In our study, we focused on CPF primarily because clinical observations have demonstrated that quinolone antibiotics can induce seizures (Slavich et al., 1989; Springuel, 1998; Kisa et al., 2005; Striano et al., 2007; Sutter et al., 2015). This phenomenon has also been corroborated in animal experiments, such as mice and zebrafish (Abdel-Zaher et al., 2012; Arafa et al., 2013; Sivarajan and Ramachandran, 2023). In fact, the research on the mechanism of antibiotic-induced epilepsy has been investigated for decades, which can be traced back to the 1990s. Here is a summary of some previous findings. First, some antibiotics have been found to inhibit GABA synthesis or block GABA receptors. β-lactams (i.e., penicillins, cephalosporins, and carbapenems) and quinolones all have been shown to act on these process (Segev et al., 1988; Hantson et al., 1999). Second, activation of excitatory transmission through activation of N-methyl-D-aspartate (NMDA) receptors, as may occur with carbapenems and quinolones (Akahane et al., 1993; Schmuck et al., 1998; Lode, 1999). Additionally, the structural similarities between certain antibiotics and epileptogenic compounds have been explored (De Sarro et al., 1989), with specific attention given to substituents at the C7 position that enhance binding to GABA receptors (Lode, 1999). Furthermore, interactions between older generation ASMs and certain antibiotics are known to influence the pharmacokinetics (Patsalos and Perucca, 2003). Coadministration of valproic acid and carbapenem will decrease the plasma valproic acid concentration by 58%, and increase the valproic acid clearance (Tobin et al., 2009). Finally, some antibiotics increase oxidative stress, potentially compromising the efficacy of epilepsy treatment (Ilgin et al., 2015; Wanleenuwat et al., 2020). However, many of these studies often induced acute and subacute seizures using large doses of CPF administered intraperitoneally, intravenously, or even intrathecally. Consequently, they may have overlooked the significant impact of CPF on the GM. Our study is the first to reveal that prolonged, clinically relevant oral administration of CPF promotes seizure susceptibility through the GM. Additionally, despite its lower BBB permeability than that of levofloxacin, CPF exhibits a higher proconvulsive potential, suggesting that its increased susceptibility to epilepsy may involve other pathways (Akahane et al., 1993). CPF is known to have a high concentration in the gastrointestinal tract (Zimmermann and Curtis, 2019), and induces gut dysbiosis (Stewardson et al., 2015; Zhu et al., 2020). Finally, considering the widespread use of CPF in the treatment of urinary tract and reproductive system infections (Thai et al., 2023), complete avoidance is challenging in certain clinical scenarios. Therefore, CPF is an appropriate instrument for studying the relationship between epilepsy and MGBA, and our study provides valuable insights for practical applications in a clinical setting.

Analysis of GM revealed a decrease in α-diversity, an estimate of species richness in CPF-14 d and Pilo group. This result is corresponding with previous animal and clinical studies (Gong et al., 2020; Oliveira et al., 2022). Decreased diversity and evenness have often been considered to reflect disease status. At the genus level, we found that some probiotic strains decreased. *Lactobacillus* can produce tryptophan and indole derivatives that regulate the immune process (Xia et al., 2021; Sun et al., 2022; Yan et al., 2022). In addition, it can modulate several neurotransmitters (Wu and Shah, 2017) and enhance the production of non-volatile acids and SCFAs (Dalile et al., 2019). Administration of probiotics, including *Lactobacillus* and *Bifidobacterium*, can reduce the seizure severity in the chronic epilepsy model induced by PTZ (Bagheri et al., 2019; Kilinc et al., 2021). Additionally, *Butyricicoccus, Parabacteroides* (Liao et al., 2024), and *Ruminococcaceae* (Peled et al., 2024) can produce SCFAs to promote health. Specifically, we found consistent changes in five genera across the two rat models: *Akkermansia, Bacteroides, Marvinbryantia, Oscillibacter*, and *Ruminococcaceae_NK4A214_group*. We observed similar findings in previous studies we observed similar findings on the GM and epilepsy (Russo, 2022; Yue et al., 2022). Among these, our particular focus was on *Akkermansia* because it ranked at the front position in the LEfSe analysis in the two rat models, which has been discussed in many previous studies. *Bacteroides* can produce SCFAs and are beneficial to the host; however, they can be opportunistic pathogens and are related to some pathological status (Shin et al., 2024). It could also be related to regulate the secretion of IL-6 and IL-17 in dendritic cells to associated with seizure severity (Xie et al., 2017). The functions of *Marvinbryantia* and *Oscillibacter* remain unclear, but they are often reported to be associated with health status or serve as protective factors (Fang et al., 2016; Xu et al., 2020; Crossland et al., 2023; Guo et al., 2023). This may be related to the production of SCFAs, which contribute to the maintenance of GM homeostasis. This suggests that these genera could be core contributors, play a substantial role in epileptogenesis, and serve as potential target genera for epilepsy treatment from the perspective of GM.

In our study, *Akkermansia* increased significantly and was ranked first in the LEfSe analysis of the two rat models. In fact, *Akkermansia* stands out as a known member of the GM and has been extensively investigated, particularly in the context of metabolic disorders and inflammatory processes (Cani et al., 2022). In studies of metabolic disorders, including obesity (Everard et al., 2014), diabetes (Plovier et al., 2017), and liver steatosis (Kim et al., 2020), *Akkermansia* has been found to be positively associated with improved metabolic health and better clinical outcomes. The same result was also observed in some inflammatory bowel diseases (Liu et al., 2021) and in response to cancer checkpoint immunotherapies (Santoni et al., 2018). Therefore, it is regarded as a paradigm for next-generation probiotics (Abuqwider et al., 2021; Cani et al., 2022). However, the “success story” of *Akkermansia* has not been without its share of controversies. In our study, the relative abundance of *Akkermansia* spanning the entire phylum significantly increased in CPF-14 d and Pilo groups. From the phylum to genus level, Verrucomicrobia, *Verrucomicrobiae, Verrucomicrobiales, Akkermansiaceae*, and *Akkermansia* displayed increased levels across all strata in the context of epilepsy. This observation is consistent with numerous studies on epilepsy (Huang et al., 2019, 2022; Arulsamy et al., 2020; Gong et al., 2021; Yue et al., 2022). Furthermore, *Akkermansia* increases have been noted in other neurological conditions, such as anxiety and depression induced by chronic stress (Li et al., 2019), multiple sclerosis (Cox et al., 2021), and Parkinson's disease (Zhao et al., 2021).

*Akkermansia muciniphila*, as the name suggests, is an exclusive mucin-degrading specialist present in the human intestine from early life (Derrien et al., 2008). *A. muciniphila* produces oligosaccharides, acetate, propionate, 1,2-propylene glycol, and ethanol via the breakdown of mucus and mucin (Derrien et al., 2004). Elevated levels of *A. muciniphila* are associated with reduced intestinal mucosal thickness, increased gut permeability, and inflammation. These factors may contribute to systemic inflammation and subsequent brain damage (Citraro et al., 2021). Previous findings suggest a potentially positive association with inflammatory status (Everard et al., 2013). The increase in *Akkermansia* in the lithium pilocarpine-induced epilepsy rat model suggests that inflammation and metabolic changes may play a role within the GM in the traditional model of chronic epilepsy. Overall, we believe that *Akkermansia* may play a complex role in the development of epilepsy, depending on the different populations and treatment choices. The relative abundance of *Akkermansia* may need to be maintained at a specific level to strike a balance between excess and deficiency.

Similar to the shared alterations observed in the GM, we conducted a comparative analysis of common changes in metabolites between the CPF and Pilo groups. Tryptophan-indole metabolism has emerged as a common phenomenon. In particular, one of the products, IPA, exhibited a consistent decreasing trend in both rat models. This change was more pronounced in the Pilo group. This discrepancy may be attributed to the fact that the lithium pilocarpine-induced epilepsy model manifests evident spontaneous seizures, whereas CPF primarily lowers the seizure threshold, representing a condition that is not strictly synonymous with an epilepsy model.

Tryptophan (TRP) metabolism pathways leading to serotonin, kynurenine, and indole derivatives are under the direct or indirect control of the GM (Agus et al., 2018). GM encodes enzymes that metabolize TRP to 5-HT, KYN, and indole metabolites, which affect TRP availability (O'Mahony et al., 2015). TRP is the only amino acid that contains an indole. In contrast to other TRP metabolites, indoles result exclusively from microbial metabolism (Kennedy et al., 2017). Indoles and their derivatives can be used as ligands to regulate inflammation and autoimmune responses *in vivo*, and play important roles in MGBA (Zhou et al., 2023b). In our studies, we have found some genera significantly related to this process (Figure 8A). These genera have been reported to be associated with metabolites involved in tryptophan metabolism, such as IPA, indoleacrylic acid (IAA), L-kynurenine, and L-tryptophan (Wikoff et al., 2009; Zelante et al., 2013; Roager and Licht, 2018; Su et al., 2022).

TRP metabolism has been explored in the context of epilepsy, with some studies focusing on kynurenic acid metabolism. We found that the concentration of kynurenic acid decreased significantly in the serum of the Pilo group, which corresponds to the findings of (Dey et al., 2022), who discovered that in a lithium-pilocarpine rat model, there was a reduction in kynurenic acid levels in the hippocampus and anterior temporal lobe. Furthermore, the exogenous application of kynurenic acid inhibits glutamatergic activity in slice preparations from rats with temporal lobe epilepsy. Promoting kynurenic acid production has been suggested as a strategy to mitigate spasms in animal models of infantile spasms (Mu et al., 2022a). In a pilot study of patients with epilepsy, tryptophan and kynurenine were shown to have the potential as diagnostic markers for epilepsy (Zhou et al., 2023a).

**Figure 9:**
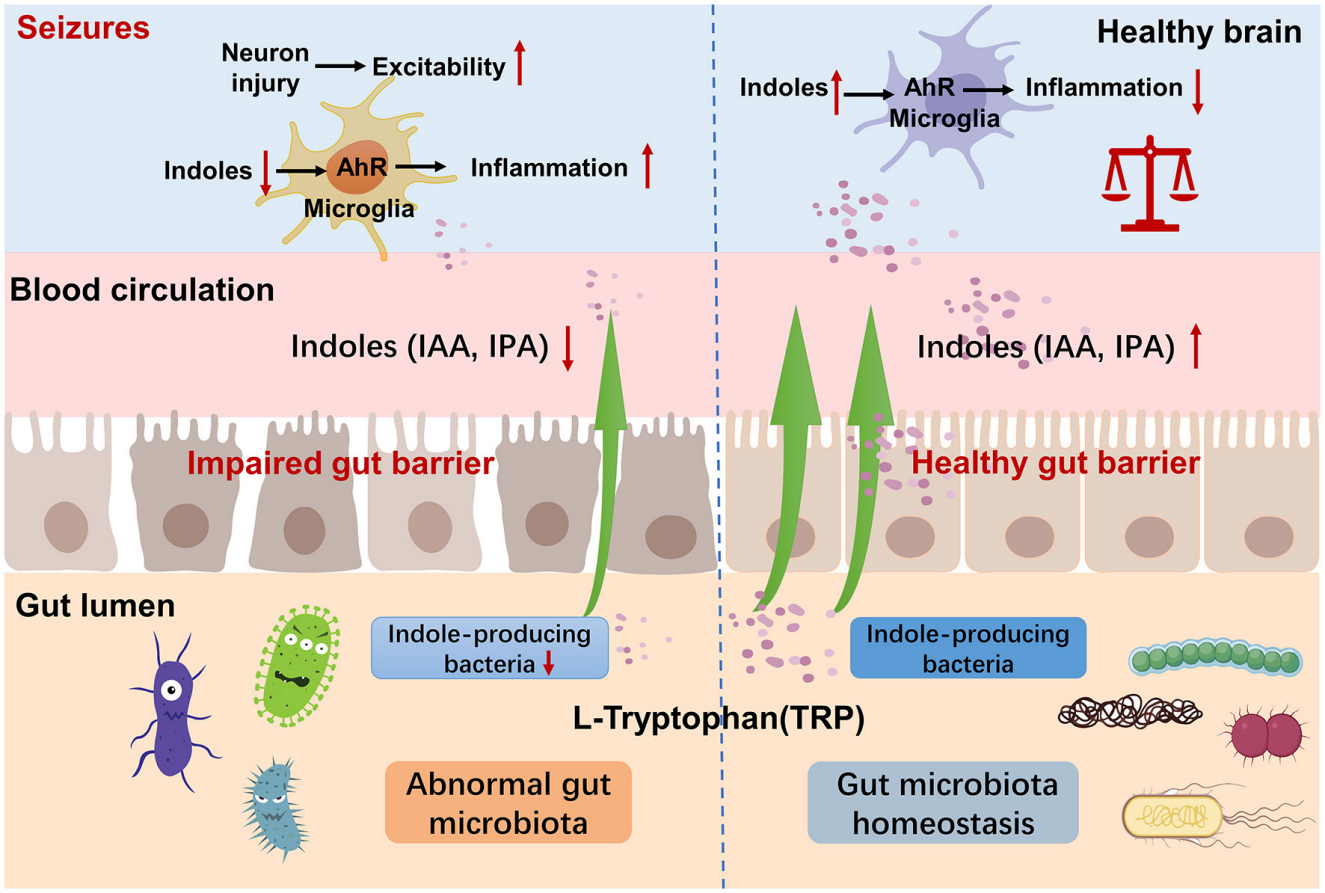
Mechanism of tryptophan-indole metabolism in neuroinflammation and epilepsy. Indole-producing bacteria regulate the AhR signaling pathway in microglia via indoles and mediated neuroinflammation.

While evidence linking epilepsy to indole and its derivatives is currently limited, a growing body of research is shedding light on their relationship with the central nervous system (CNS), which serves as a neuroprotective factor. In 1980, Young et al. (1980) conducted an experiment on rats, indicating that IPA in the CSF is not derived from the CNS, but from bacterial metabolism in the gut. It has a high permeability to the BBB and does not exhibit a gradient. IPA is a small molecule of indoles that can passing through the lipid bilayer via free diffusion (Piñero-Fernandez et al., 2011). IPA was detected in the gut, serum, and brain of SPF mice compared to germ-free mice, thereby its production was interpreted to be fully mediated by the GM (Sarkar et al., 2016; Loh et al., 2024). The GM affects the diversity of indole compounds in serum, and GM induction is sufficient to introduce IPA into the host (Wikoff et al., 2009). Probiotics increase the level of IPA, reduce the inflammatory process, lower the HPA axis regulation factors, and reduce depressive-like behavior (Abildgaard et al., 2017). All of these showed a close correlation between the GM and IPA. In CNS studies, plasma IPA levels were significantly lower in patients with Huntington's disease (Rosas et al., 2015). Liu et al. (2020) found that IPA and 5-HT treatment significantly attenuated cognitive deficits in diabetic mice. In Alzheimer's disease (AD), IPA can protect against Aβ-induced neuronal death as a scavenger of free radicals (Chyan et al., 1999). Sun et al. (2022) found that the indoles derivates inhibited the activation of the NF-κB signal pathway as well as the formation of the NLRP3 inflammasome, reduced the release of inflammatory cytokines, and then reduced the neuroinflammation in AD transgenic mice. Xie et al. (2022) found that the administration of IPA attenuated the activity of neurotoxic reactive A1 astrocytes in a mouse model of ischemic stroke. And IPA protects neurons from ischemia-induced damage by reducing DNA damage and lipid peroxidation (Hwang et al., 2009). In recent years, it has become evident that indole derivates, including IAA and IPA, are acting as ligands of the aryl hydrocarbon receptor (AHR). AHR is a transcription factor extensively expressed in astrocytes and microglia (Gutiérrez-Vázquez and Quintana, 2018). Its activation leads to alterations in both the innate and adaptive immune responses, subsequently regulating intestinal epithelial function, gut barrier integrity, and GM composition (Dong et al., 2020; Scott et al., 2020; Li et al., 2021). Previous study on multiple sclerosis mouse model showed that AHR acts as a negative regulator of NF-κB activation (Rothhammer et al., 2016). In recent years, more and more researchers have confirmed that neuroimmunity and inflammation also plays a crucial role in epilepsy (Vezzani et al., 2011, 2015). The changes in GM and tryptophan-indole metabolism mentioned above may collectively contribute to the development of epilepsy (Figure 9).

### 4.1 Limitations

The utilization of animal models in this study poses limitations when extrapolating the findings to human conditions. Due to the differences of species, the translatability of results from animal models to humans, especially concerning complex biological processes such as GM interactions, demands cautious interpretation and may not fully encompass the intricacies of human physiology.

## 5 Conclusions and future directions

In this study, we presented new evidence for the first time that CPF can directly alter GM, leading to increased epilepsy susceptibility in rats. The reversal of FMT on the susceptibility to epilepsy confirmed that GM and MGBA may be pivotal mechanism underlying the increased susceptibility to epilepsy induced by CPF. Changes in the genera *Akkermansia, Bacteroides, Marvinbryantia, Oscillibacter*, and *Ruminococcaceae_NK4A214_group* have been shown to be associated with epileptic activity in two epilepsy related animal models. In addition, GM-produced amino acid metabolites may be critical to the mechanisms of MGBA in relation to seizures and epilepsy. IPA, a product of tryptophan-indoles metabolism, may play a crucial role in this process from the perspective of neuroimmune regulation.

In future research on the mechanisms or clinical translation potentials referring the GM and epilepsy, a starting point could be the core GM genera or the tryptophan-indole metabolic pathway mentioned above. For instance, modulation of the relative abundance of core GM genera through probiotics or targeted drugs, or supplementation of tryptophan metabolites such as kynurenic acid and IPA, could be investigated. Further molecular mechanism studies are necessary to elucidate how GM influence seizure susceptibility or the development of epilepsy, and to identify molecular-level treatment targets.

## Data availability statement

The original contributions presented in the study are publicly available. This data can be found here: NCBI BioProject, accession PRJNA1056293, PRJNA1056516.

## Ethics statement

The animal study was approved by the Institutional Ethical Committee for Animal Welfare of the Seventh Affiliated Hospital of Sun Yat-sen University. The study was conducted in accordance with the local legislation and institutional requirements.

## Author contributions

SZ: Conceptualization, Data curation, Formal analysis, Investigation, Methodology, Software, Supervision, Writing – original draft, Writing – review & editing. YL: Conceptualization, Data curation, Methodology, Supervision, Writing – review & editing. QZ: Data curation, Investigation, Methodology, Writing – review & editing. MY: Data curation, Methodology, Writing – review & editing. HLi: Conceptualization, Formal analysis, Investigation, Methodology, Project administration, Writing – review & editing. RN: Formal analysis, Methodology, Writing – review & editing. HLa: Formal analysis, Supervision, Writing – review & editing. JW: Methodology, Writing – review & editing. XY: Conceptualization, Funding acquisition, Resources, Supervision, Validation, Visualization, Writing – review & editing. LZ: Conceptualization, Funding acquisition, Investigation, Resources, Supervision, Validation, Writing – review & editing.
